# Supplementary material for: Different Transcutaneous Auricular Vagus Nerve Stimulation Parameters Modulate the Anti-Inflammatory Effects on Lipopolysaccharide-Induced Acute Inflammation in Mice
Source: Biomedicines. 2022 Jan 24;10(2):247. doi: 10.3390/biomedicines10020247 (PMC8869637; doi:10.3390/biomedicines10020247)
Supplement: Supplementary file 1 [file biomedicines-10-00247-s001.zip › Supplementary Figures.pdf]

Different transcutaneous auricular vagus nerve stimulation parameters modulate the anti-inflammatory effects on lipopolysaccharide-induced acute inflammation in mice

Yoon Young Go, PhD[a,b], Won Min Ju[a], Chan Mi Lee[a], Sung-Won Chae, MD[a,b], Jae-Jun Song, MD[a,b,c]*

[a]Department of Otorhinolaryngology- Head and Neck Surgery, Korea University Guro Hospital, Seoul 08308, Korea

[b]Institute for Health Care Convergence Center, Korea University Guro Hospital, Seoul 08308, Korea

[c]Neurive institute, Neurive Co.Ltd., Seoul 08308, Korea.

*Corresponding author:

Jae-Jun Song, MD/PhD

Department of Otorhinolaryngology- Head and Neck Surgery,

Korea University Guro Hospital,

80 Guro-dong, Guro-gu,

Seoul 08308, South Korea

Tel: 82-2-2626-3186; Fax: 82-2-2626-0475; E-mail: jjsong23@gmail.com

Supplementary materials

Supplementary video. taVNS 15 Hz and 25 Hz

These videos show the taVNS treated mice in two different pulse frequencies of taVNS (15 Hz and 25 Hz).

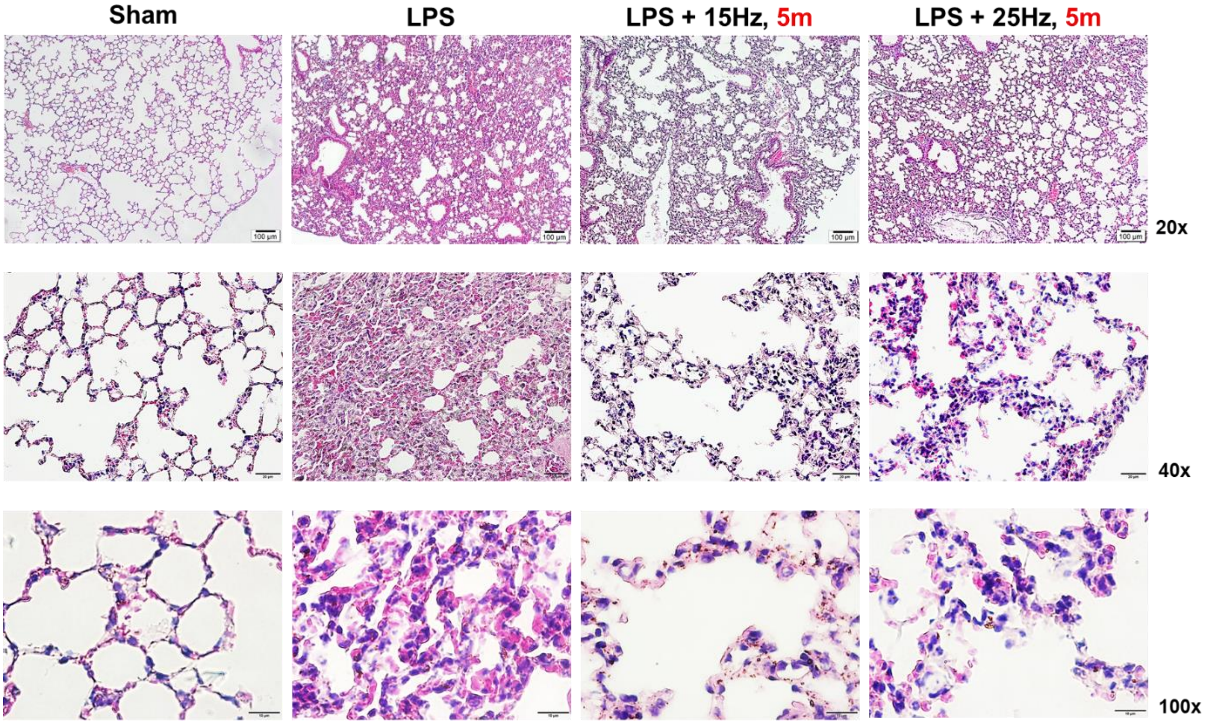

Supplementary Figure S1. Histological evaluation of the lung in taVNS 15 Hz for 5 min and 25 Hz for 5 min on LPS-induced endotoxemia

Hematoxylin and eosin (H&E) staining results of the lung in taVNS treatment with 15 Hz for 5 min and 25 Hz for 5 min on LPS-induced inflammation. Scale bar: 100 μm (20x), 20 μm (40x), 10 μm (100x), respectively.

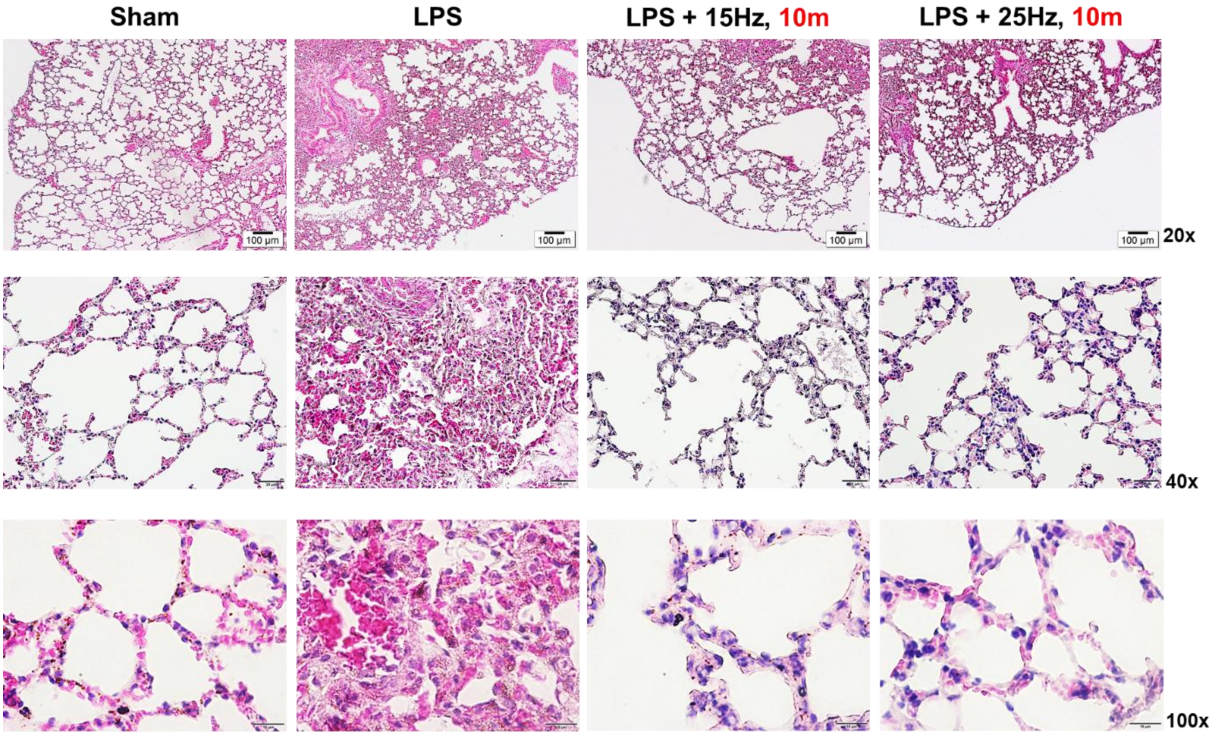

Supplementary Figure S2. Histological evaluation of the lung in taVNS 15 Hz for 10 min and 25 Hz for 10 min on LPS-induced endotoxemia.

Hematoxylin and eosin (H&E) staining results of the lung in taVNS treatment with 15 Hz for 10 min and 25 Hz for 10 min on LPS-induced inflammation. Scale bar: 100 μm (20x), 20 μm (40x), 10 μm (100x), respectively.

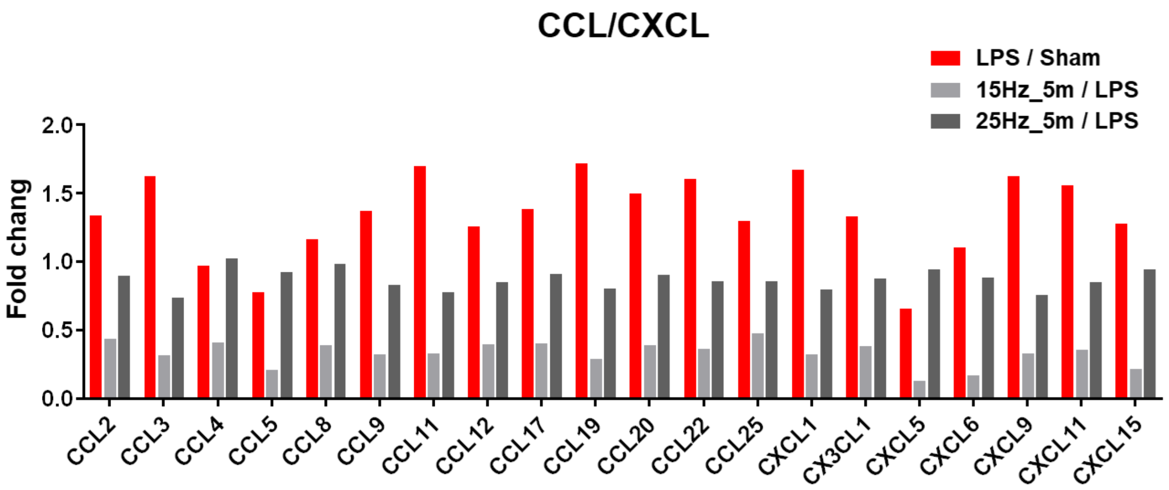

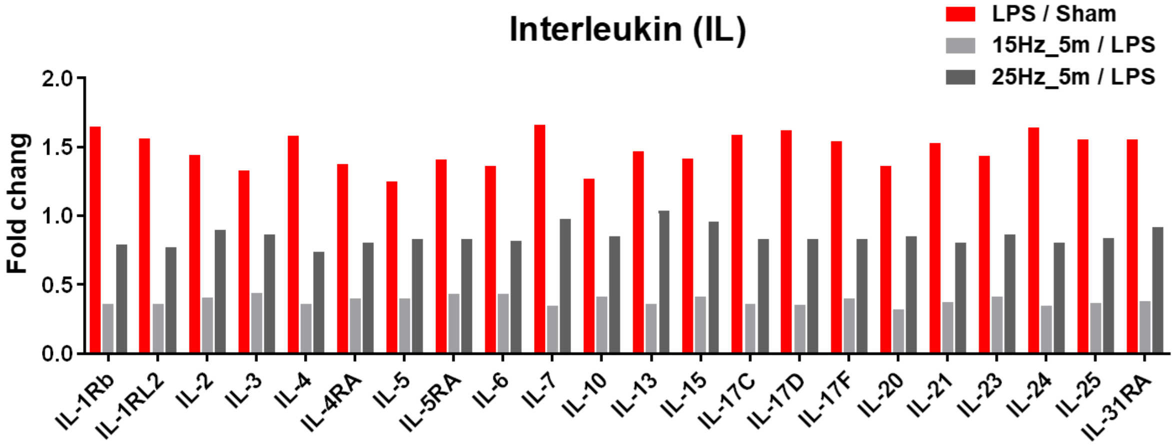

Supplementary Figure S3. Fold changes of CCL/CXCL/IL in 15 Hz and 25 Hz taVNS on endotoxemia mice.

The relative expression of CCL/CXCL/IL in 15 Hz and 25 Hz taVNS on LPS-induced inflammation were compared using fold changes.

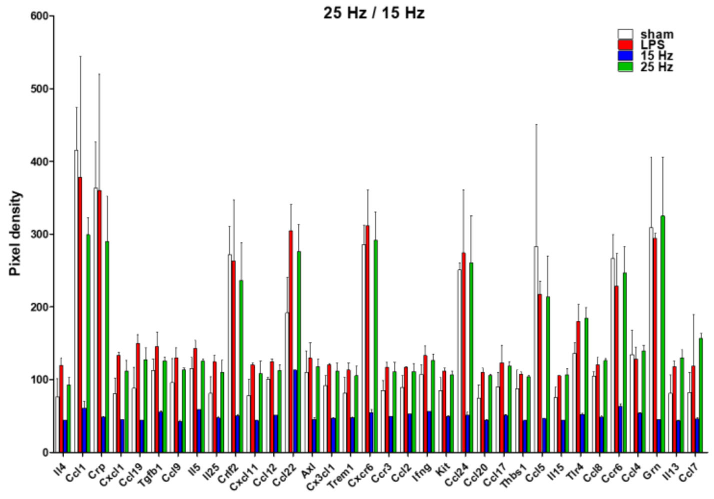

	Sham		LPS		15 Hz		25 Hz	
**Il4**	94.0	58.5	127.0	111.5	44.5	44.0	85.5	100.0
**Ccl1**	373.0	457.0	495.5	260.5	67.5	54.0	315.5	282.5
**Crp**	318.5	408.0	473.0	247.5	49.5	48.0	333.5	246.0
**Cxcl1**	96.0	65.5	130.0	136.5	45.0	44.5	102.0	122.0
**Ccl19**	108.5	67.5	158.5	141.0	44.5	44.0	115.0	139.0
**Tgfb1**	123.5	101.0	159.5	131.0	54.0	56.5	122.0	129.5
**Ccl9**	119.5	72.0	140.0	120.0	42.0	43.5	115.5	112.0
**Il5**	126.5	104.5	151.0	134.0	59.0	59.0	123.0	127.5
**Il25**	97.5	64.5	131.0	118.0	48.5	46.0	98.5	122.0
**Crlf2**	299.0	244.5	322.5	204.0	48.5	51.5	273.0	199.5
**Cxcl11**	94.0	62.5	122.0	118.5	43.5	44.0	96.0	120.5
**Ccl12**	99.0	102.5	127.0	122.0	51.0	51.0	106.5	118.0
**Ccl22**	226.0	157.5	330.5	279.0	113.5	113.0	249.5	302.5
**Axl**	130.5	89.5	144.5	114.5	47.0	44.0	125.0	110.5
**Cx3cl1**	101.5	81.5	121.5	119.0	47.5	46.5	104.0	119.5
**Trem1**	96.5	66.5	120.0	106.0	48.0	46.5	96.0	114.5
**Cxcr6**	304.5	267.0	346.5	276.0	57.5	52.5	319.0	264.0
**Ccr3**	95.0	75.0	112.5	122.0	49.5	49.5	101.5	120.0
**Ccl2**	100.5	77.0	117.5	117.0	53.0	52.5	103.5	119.0
**Ifng**	116.5	97.5	142.5	123.5	56.5	56.0	119.5	132.5
**Kit**	97.5	72.0	115.0	109.0	48.5	50.0	102.5	110.5
**Ccl24**	257.5	244.0	335.5	212.5	54.0	49.0	306.5	215.0
**Ccl20**	87.5	62.0	114.0	106.5	45.0	44.0	105.0	107.0
**Ccl17**	104.0	76.0	140.0	106.5	50.0	51.5	123.0	114.0
**Thbs1**	105.5	69.5	104.5	110.0	44.0	43.5	103.5	105.0
**Ccl5**	401.5	165.0	230.0	205.0	46.0	47.0	253.5	174.5
**Il15**	85.5	65.0	105.5	105.0	44.5	44.5	100.5	112.5
**Tlr4**	146.5	126.0	196.5	163.0	51.0	53.0	195.0	174.0
**Ccl8**	109.0	101.0	127.5	113.5	50.0	47.0	128.0	124.0
**Ccr6**	289.5	243.0	196.0	260.5	65.5	61.0	272.5	220.5
**Ccl4**	158.0	111.0	116.5	139.5	54.5	54.0	145.0	134.5
**Grn**	377.0	240.5	289.0	299.0	45.0	45.0	382.0	268.0
**Il13**	99.0	63.5	123.0	112.5	44.0	42.5	137.5	122.0
**Ccl7**	102.0	62.5	68.5	169.0	47.0	45.0	152.5	161.5

Supplementary Figure S4. The pixel intensity of chemokines/cytokines in 15 Hz and 25 Hz taVNS on endotoxemia mice.

The significantly downregulated chemokines/cytokines are listed in the graph and table.
